# Theory, Prognosis and Treatment of Epilepsy

**Published:** 1886-11

**Authors:** 


### Editorial.

### *THEORY, PROGNOSIS AND TREATMENT OF EPILEPSY.*

In 1881, Dr. Gowers, of London, published a most remarkable and comprehensive treatise on epilepsy; he reviewed at considerable length, the various theories of epilepsy, and arrived at the conclusion that the theory of Hughlings-Jackson was the only one which could explain the phenomena of epilepsy. This theory has but few adherents at the present time, and one need but hear a discussion on this subject to see how little understood it still is.

This is greatly to be regretted, for, based on it, we have a plan of treatment which has proven of inestimable value to the profession, and has taken epilepsy from among the utterly hopeless diseases and placed it among the curable. Gowers demonstrated, by the treatment of a great many cases, that this was correct.

It has been substantiated by many who have given it diligent study and carefully followed the most minute directions.

The theory of Hughlings-Jackson is that of a discharging lesion of the cells of the gray matter of the brain. That it is

"in most cases within the cerebral hemispheres, probably often in the cerebral cortex although possibly, in some instances lower down and may be even in the medulla." "What is the nature of this tissue change? Can we form any opinion as to the character of the alteration in the gray matter which permits its sudden discharge? It is answered in the following language: 'In the construction of a motor nerve center, it is requisite to create an apparatus which is always ready to evolve force, but which shall not do so spontaneously without the application of a stimulus of some kind or other, either physical or mental. The peculiarity of nerve cells is that they possess two qualities: They prepare material which, by undergoing oxidation, generates force, and yet they can prevent this material from so acting, although blood is circulating all round it charged with oxygen. In states of spasm this property of the nerve cell is lost or much impaired, and the discharge of highly unstable cells leads to the discharge of healthy cells in other centers, with which there is an anatomical connection by fibres. The discharging lesion may be likened to a fulminate which overcomes the resistance of less unstable compounds. The nerve cells are likened to so many Leyden jars, which generate the electricity. Thus the explosion is due to a sudden diminution of resistance by which the pent-up nerve force is released, thus causing the epileptic phenomena.'"

Enough as to the theory; as to prognosis we find that it "involves several separate questions: (1) The danger to life, (2) the prospect of a spontaneous termination of the disease, (3) the prospect that by treatment the disease may be (a) cured, or (b) the attacks arrested." We might add a 4th; the prospect of arresting mental decay, where we do not completely arrest the attacks. As to the first we find there is such a slight danger to life that we will not take the space to discuss it; 2d, the spontaneous termination of the disease; but few cases terminate spontaneously and these are usually patients under five years of age. They frequently suffer from the trouble in later life, and are not so apt to be free from it as those who are relieved by treatment, and that the mental impairment is far greater. As to "*the probability of cure or arrest by treatment,*" the only method of cure is by obtaining arrest of the fits for a considerable time." The indications of the prognosis has been materially changed by the introduction of the bromides as remedies for epilepsy. Not only do they arrest fits far more frequently than any other remedy, but they are effective in many cases, which, according to experience, previous to the introduction of these remedies, would have been regarded as most unpromising. As to age it was advanced by Gowers that "the proportion of the cases commencing under twenty, in which arrest was obtained, is considerably less than the proportion of cases commencing over twenty. The difference amounting to about 13 per cent." As to duration before treatment was instituted, "the result is in agreement with the conclusions of all writers on the subject, that the prognosis is favorable in inverse proportion to the duration of the disease." While this is undoubtedly true, we must not allow it to interfere with our plan, or influence us in treating the disease. A case of twelve year's standing, well known to the writer, was relieved in four months, and after two years the attacks have not returned. Many other cases of similar character could be cited.

Heredity plays but a small, if any, part in rendering the prognosis less favorable. We see then that many cases are completely cured; that no class of cases can be called unpromising, unless there be complete or partial dementia; indicative of brain change having taken place.

As to the fourth, we find that the mental decay, so often seen in this disease, does not follow in cases treated from time to time by the use of the bromides, or where patients take moderate doses of this drug, even if the attacks be not completely arrested.

As to the treatment, we must pay particular attention to the general condition of our patient. In many cases it being of great service to precede the use of the bromides by tonics, being careful in all cases to relieve constipation, and, so far as in our power, to improve the condition of the digestive tract. When the bromides are given to bring our patient rapidly under its influence, producing within a week, or a comparatively short time, slight bromism, to then reduce the remedy to the minimum doses. The usual amount required being ʒi. to ʒii. of the drug daily, giving it in four divided doses, taking care to administer one dose during the night. The drug should never be suspended; should be continued for fourteen to eighteen months after the cessation of the attacks. In many cases the combination of the three bromides of ammonium, potassium and sodium will act more kindly than any one alone. By far the most useful of any single bromide is the bromide of potassium.

Many drugs do great good, if associated with the bromides and chief among these is the iodide of potassium. Where syphilis is suspected it is of course necessary, but where it is not, many cases have undoubtedly been greatly benefitted by its use, Dr. Salter, in speaking of its great utility in the treatment of various neuroses says: "Of its ultimate and exact modus operandi, I can neither offer an explanation, nor form any reasonable opinion." It should be given in small doses and continued for months. We will find ergot of great service in many cases. Digitalis, iron, belladona, all have their place as associate drugs. We must even keep in mind the proper regulation of the diet. We must place our patient under the best hygienic conditions, to accomplish this we will frequently find it necessary to send our patients to a hospital, putting them under the strictest orders, as to diet, exercise and regular habits. There are many cases that could be reported to illustrate this point. Thus it is to be hoped the day is not far distant when a hospital, both public and private, will be established to assist in the treatment of proper cases. As to many other remedies suggested and used we will find the following of value:

"Dr. C. L. Dana, has written a paper, which is published in a recent number of the *New York Medical Journal.* His conclusions are summarized in the following statements:—

"I. Diet, exercise and proper hygienic treatment rank above all other single therapeutic measures.

"II. The bromides take the second rank in the treatment of epilepsy.

"All bromides act alike in this disease. If one does not cure another will not. Occasionally changing and mixing reduces the attacks for a time, and benefits the stomach.

"III. The best bromides are those of potassium, sodium, ammonium, and hydrogen (hydrobromic acid); possibly we may add nickel.

"IV. Bromides may be given in daily doses of ʒj, increased gradually until the attacks are suppressed, or the dose reaches ʒiv to ℥j daily. Few patients can tolerate more than this latter dose. Thorough bromidization should be always tried, if necessary to stop fits, and it may be occasionally repeated; but bromidization is sometimes injurious, even making the disease worse, and it must always be employed with caution.

"V. When the fits are suppressed, the bromides should be carefully reduced, but never entirely stopped for at least two years after the last fit.

"VI. In most cases, and especially in nocturnal epilepsy, an extra large dose of bromide should be given at night.

"VII. It is very important that bromides should be chemically pure, that their use should be continued a very long time, and that their depressing effects should be offset by tonics and all possible roborant measures.

"VIII. The best non-specific adjuvants (drugs) to the bromides are potassium iodide, (in syphilitic epilepsy,) potassium bicarbonate, (in lithæmic and rheumatic states), carbonate of ammonium, the hypophosphites, arsenic, iron and quinine

"IX. The other chief adjuvants to the bromides are diet, exercise, a regular life, hydrotheraphy, counter-irritation on the neck, and, in the line of drugs, zinc, belladona, strychnine, valerian and the nitres. Combinations of bromides with the other drugs mentioned will lessen attacks when bromides alone will not.

"X. The best substitutes for the bromides, when these do no good or do harm, are belladonna, zinc, strychnine, glonoin, borax and alteratives.

"For nocturnal epilepsy, increase the dose of bromide at night, and add chloral or digitalis. Give also, if needed, strychnine. Raising the head of the bed or making the patient sleep in a chair at night are measures to be tried.

"For the status epilepticus give large enemata of chloral, and use emetics and purges. Venesection is often efficacious, morphine is dangerous, chloroform is only palliative, and nitrite of amyl is of little value."—*Boston Medical and Surgical Journal.*

